# Qianjin Baoyun Wan reduces spontaneous abortion and the prothrombotic state in pregnant rats

**DOI:** 10.1016/j.jtcme.2026.09.006

**Published:** 2026-09-23

**Authors:** Hekun Zeng, Hui Ding, Tingting Wang, Mengqin Yi, Anzidine Mohamed, Jiaqin Pan, Mingyao You, Qunqun Du, Anqi Luo, Renan Qin, Hong Nie

**Affiliations:** a State Key Laboratory of Bioactive Molecules and Druggability Assessment, Guangdong Basic Research Center of Excellence for Natural Bioactive Molecules and Discovery of Innovative Drugs/International Cooperative Laboratory of Traditional Chinese Medicine Modernization and Innovative Drug Development of Ministry of Education (MOE) of China/Guangdong-Hong Kong-Macau Joint Laboratory for Pharmacodynamic Constituents of TCM and New Drugs Research, College of Pharmacy, Jinan University, Guangzhou, Guangdong, 510632, China; b Guangzhou Baiyunshan Chenliji Pharmaceutical Factory Co., Ltd., Guangzhou, 510288, China; c Guangzhou Pharmaceutical Co. Ltd., Guangzhou, 510130, China; d Department of Neurology, The Affiliated Hospital of Guizhou Medical University, Guizhou, China

**Keywords:** Qianjin Baoyun Wan, Prothrombotic state, Antithrombotic, Recurrent spontaneous abortion, Traditional Chinese medicine

## Abstract

**Background and aim:**

Recurrent spontaneous abortion (RSA) is closely associated with a prothrombotic state (PTS) in the first trimester of pregnancy. Qianjin Baoyun Wan (QBW), a traditional Chinese medicine formula, has historically been prescribed to prevent threatened miscarriage and pregnancy-related lumbago, while its efficacy and underlying mechanisms in RSA complicated by PTS remain to be elucidated. This study aimed to investigate the therapeutic effects and potential pharmacological mechanisms of QBW in a rat model of RSA with PTS.

**Experimental procedure:**

Spontaneous abortion was induced in pregnant rats by mifepristone, and PTS was established via epinephrine administration. The therapeutic effects of QBW were assessed by measuring miscarriage rate, uterine contractility, and uterine microcirculation morphology. Coagulation-related parameters (APTT, TT, PT, fibrinogen) were evaluated. Thrombosis-related and pregnancy-related biomarkers were quantified by ELISA. In addition, network pharmacology and network toxicology were employed to predict the potential pharmacodynamic targets and toxicological profiles of QBW.

**Results and conclusion:**

QBW reduced miscarriage rates and uterine contractility while improving pregnancy-related biomarkers. It restored uterine microcirculation (capillary density and diameter), prolonged coagulation time, lowered fibrinogen, and restored the balance between antithrombotic factors (e.g., AT-III, 6-keto-PGF_1α_) and prothrombotic factors (e.g., D-dimer, TXB_2_). Network pharmacology predicted immunoregulatory, anti-inflammatory, and antioxidant mechanisms underlying its effects against RSA with PTS, though potential signals of hepatic and reproductive organ effects were noted. These findings suggest that QBW may have therapeutic potential for RSA with PTS, although further preclinical and clinical validation is required.

## Highlights

• QBW reduced miscarriage number and rate in rat model of RSA with PTS.
• QBW reduced uterine hypercontraction and restored its microcirculation.
• QBW prolonged coagulation time and lowered fibrinogen levels.
• QBW rebalanced prothrombotic and antithrombotic factors in serum.
• Network analysis predicted multiple therapeutic mechanisms of QBW.

## Graphical abstract

**Figure:**
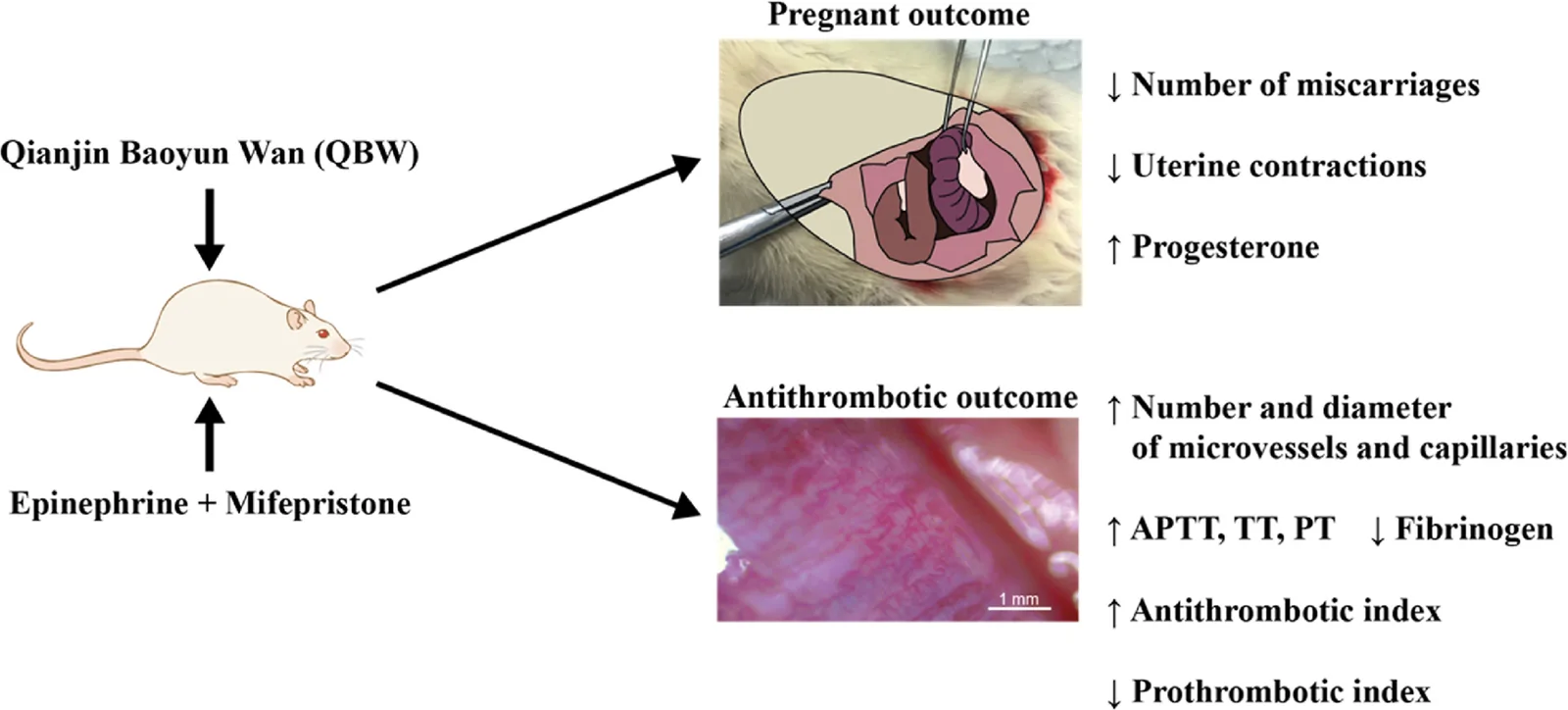

## Abbreviations:

RSA: recurrent spontaneous abortion
PTS: prothrombotic state
QBW: Qianjin Baoyun Wan
LDA: low-dose aspirin
LMWH: low-molecular-weight heparin
APTT: activated partial thromboplastin time;
PT: prothrombin time;
TT: thrombin time;
β-CG: chorionic gonadotropin β
AT-III: antithrombin III
6-keto-PGF_1α_: 6-keto-prostaglandin F_1α_
TXA_2_: thromboxane A_2_
TXB_2_: thromboxane B_2_
F1+2: prothrombin fragment 1 + 2
ELISA: enzyme-linked immunosorbent assay
IL: interleukin
TCM: Traditional Chinese Medicine;
OB: oral bioavailability
DL: drug-likeness
GO: Gene Ontology
BP: Biological Process
CC: Cellular Component
MF: Molecular Function
PR: Progesterone Receptor
eNOS: endothelial nitric oxide synthase
NO: nitric oxide;
EGFR: epidermal growth factor receptor
VEGF: vascular endothelial growth factor
StAR: steroidogenic acute regulatory protein

## 1 Introduction

Recurrent spontaneous abortion (RSA), or recurrent pregnancy loss, is defined as 3 consecutive miscarriages or fetal loss before the 20th week of pregnancy, affecting approximately 1-3% of women of reproductive age.^1^ A prothrombotic state (PTS), particularly of autoimmune origin, is found in 5-20% of RSA patients and promotes placental thrombosis, complement activation, and inflammation, ultimately leading to fetal loss through hypoxia and ischemia.^2^ PTS in pregnancy also arises from physiological hypercoagulability and has been linked to gestational diabetes, preeclampsia, and proteinuria, underscoring that its management in RSA patients serves both fetal and maternal health.^3^, ^4^, ^5^.

The pathophysiology of RSA with PTS extends beyond thrombosis alone and involves a complex interplay between endocrine insufficiency and uteroplacental vascular dysfunction. Progesterone deficiency disrupts decidual support and trophoblast invasion, while prothrombotic microcirculatory impairment compromises uteroplacental perfusion, creating a convergent pathological axis at the maternal-fetal interface, unfavorable for normal trophoblast function.^6^, ^7^, ^8^, ^9^ Adrenergic activation represents one mechanistic pathway capable of aggravating such vascular dysfunction through effects on vascular tone, endothelial activity, and coagulation, as supported by murine studies linking excessive epinephrine signaling to early pregnancy loss.^10^

To experimentally capture the interaction between hormonal insufficiency and vascular dysregulation, we employed a combined rat model integrating mifepristone-induced progesterone withdrawal with epinephrine-mediated PTS. This dual-hit design was motivated by the clinical observation that PTS acts as a contributing factor rather than a sufficient cause of pregnancy loss, and its convergence with progesterone insufficiency represents a clinically relevant high-risk combination that single-factor models cannot capture.^11^, ^12^, ^13^.

To prevent RSA or to protect pregnant women with PTS from fetal loss, low-dose aspirin (LDA) and low-molecular-weight heparin (LMWH) are often prescribed as antiplatelet or anticoagulation agents to prevent thrombosis in the clinic. Although meta-analyses support anticoagulation in antiphospholipid antibody-positive patients, large randomized trials showed no significant improvement in live births for unexplained RSA or inherited thrombophilia.^14^, ^15^, ^16^ Hence, novel drugs are needed, and Traditional Chinese Medicine (TCM) formulae have been suggested as potential candidates for threatened miscarriage.^17^

Qianjin Baoyun Wan (QBW) is a TCM formula recorded in the Pharmaceutical Standard of the Ministry of Public Health of the People's Republic of China-Traditional Chinese Patented Medicine (No. WS3-B-0183-90), to treat the excessive fetal movement, vaginal bleeding, and lumbago, during pregnancy and to prevent miscarriage. It is derived from Xionggui Jiaoai Decoction (XGJAD, or Xiong-gui-jiao-ai-tang in Mandarin Chinese, kyuki-kyogai-to in Japanese), a classical formula recorded in Zhang Zhongjing's Jingui Yaolüe (Eastern Han Dynasty) for “nourishing blood to calm the fetus” and proven effective in treating threatened abortion in early pregnancy and intestinal bleeding.^18^^,^^19^ However, experimental evidence on QBW in spontaneous abortion and PTS is lacking, highlighting the need to validate the effectiveness and mechanism in animal models.

Therefore, the present study evaluated whether QBW could ameliorate miscarriage in RSA complicated by PTS by assessing pregnancy outcomes, uterine contraction, uterine microcirculation, coagulation parameters, and endocrine homeostasis, with network pharmacology and toxicology analyses to predict its mechanism and safety profile.

## 2 Materials and methods

### 2.1 Animal

71 pregnant SPF Sprague-Dawley female rats (8 weeks old, 250 ± 20 g, on days 1-3 of gestation) were purchased from the Zhuhai Bestest Biotechnology Co., Ltd. (Certificate No. SCXK (Guangdong) 2020-0051). All rats were housed under a 12 h light/dark cycle and given standard laboratory food and tap water at the Institute of Biological and Medical Engineering, Guangdong Academy of Sciences (Certificate No. SYXK (Guangdong) 2023-0183).

### 2.2 Establishment of PTS and spontaneous abortion pregnant rat model and drug administration

The model was established as previously described.^20^^,^^21^ In brief, 71 pregnant rats were randomized into 6 groups: control (n = 11), model, aspirin (6.75 mg/kg), and QBW at 0.88, 1.76, and 3.52 g/kg (n = 12 each). All groups except the control received subcutaneous epinephrine hydrochloride (0.8 mg/kg, once daily on days 0-10) to induce PTS, followed by intragastric (i.g.) mifepristone (4 mg/kg) on day 10 at 30 min before sacrifice to induce spontaneous abortion (Supplementary Fig. S1). Aspirin (dissolved in saline) or QBW (suspended in saline) was administered i.g. at 8 h after each daily epinephrine injection. QBW doses were set using the body surface area formula with geometric escalation, corresponding to 0.5×, 1×, and 2× the daily human equivalent dose (∼70 kg), to explore dose-dependence while reducing animal usage. The highest dose was within the range commonly used in rodent TCM studies, and no toxicity or mortality was observed. Reagents: epinephrine hydrochloride (Lot#220314, Shanghai Full Woo Biotechnology, Zhumadian, China); mifepristone (Lot#M2134083) and aspirin (Lot#I2122015) (Shanghai Aladdin Bio-Chem Technology, Shanghai, China); QBW (Lot#A52019, Guangzhou Baiyunshan Chenliji Pharmaceutical Factory, Guangzhou, China; purchased from a local pharmacy).

### 2.3 Quantitative examination of miscarriages

At 30 min after the last drug administration on day 10, rats were anesthetized intraperitoneally with 30 mg/kg pentobarbitone, fixed to open the lower abdomen to remove the uterus. Fetuses with their placentas were identified and counted. The surviving fetuses were defined as having a bright pink color with clear contours and no hemorrhagic stains, while the lost fetuses were defined as having a decreased size, hemorrhage and necrosis of the placenta, and petechial hemorrhages in the fetus. The number of miscarriages was calculated as placentas minus surviving fetuses, and rate of miscarriage as miscarriages divided by placentas.

### 2.4 *In vivo* examination of uterine contraction

After anesthetization, the upper third of one uterine horn was isolated in situ without transection. A surgical thread was passed beneath the segment, and its two ends were tied to the two ends of a wooden stick (∼2 cm in length) placed adjacent to the uterus. The midpoint of the thread was then connected, via a second surgical thread, to the force transducer of the BL-420N DataLAB data acquisition system (Chengdu Techman Co., Ltd., Chengdu, China) to record isometric uterine contractions. After applying a preload of −2 g followed by 15 min calibration at 0 g, uterine contractions were recorded for 1 h, and the peak contraction force, contraction frequency, and contraction intensity (area under the curve) were calculated.

### 2.5 Observation and measurement of uterine microcirculation

After anesthetization, rats were fixed supine on a 37 °C heating pad. A surgical incision was made along the midline of the abdomen to expose the uterine horns for direct *in vivo* observation. During observation, the uterine surface was moistened with saline pre-warmed to 37 °C to prevent drying. The perimetrium was visualized using a 60× stereomicroscope, and images were continuously recorded on a laptop for 30 min to identify capillaries (≤10 μm in diameter) and microvessels (11-100 μm in diameter). The number of vessels crossed by a midline drawn along the perimetrium was counted, and the diameter of each vessel was measured near its crossing point with the midline, as shown in Supplementary Fig. S2. Data were collected at 10, 20, and 30 min after the start of the recording. All images were analyzed by two trained independent observers blinded to the group allocation, and the average values were calculated for statistical analysis.

### 2.6 Quantitative evaluation of relative organ weight

After anesthetic overdose, the rats were weighed and sacrificed, and the uterus, ovaries, kidneys and spleen were removed, briefly cleaned with saline and dried with tissue. The uterus, ovaries, kidneys and spleen were weighed and the relative organ weights (organ coefficient) were calculated as the ratio of organ weight to body weight.

### 2.7 Evaluation on coagulation-related parameters

Blood was collected from the abdominal aorta via vacuum blood-collecting tubes containing sodium citrate anticoagulant. Plasma was obtained by centrifugation (2500 rpm, 15 min, 4 °C), and activated partial thromboplastin time (APTT), prothrombin time (PT), thrombin time (TT), and fibrinogen were measured on an automatic coagulation analyzer (ACL Top 700, Instrumentation Laboratory, USA) following the standard protocol.

### 2.8 Enzyme-linked immunosorbent assay (ELISA) for thrombosis and pregnancy related factors (hormones)

Blood from the abdominal aorta was allowed to clot spontaneously, and serum was collected after centrifugation (2500 rpm, 10 min, 4 °C) and stored at −80 °C. The concentrations of estradiol, progesterone, chorionic gonadotropin β (β-CG), antithrombin III (AT-III), 6-keto-prostaglandin F_1α_ (6-keto-PGF_1α_), prostacyclin, D-dimer, thromboxane A_2_ (TXA_2_), thromboxane B_2_ (TXB_2_), and prothrombin fragment 1 + 2 (F1+2) were analyzed by ELISA kits (Lot#202207, Jiangsu Meimian Industrial Co., Ltd., Yancheng, China) following the manufacturer's protocol.

### 2.9 Network pharmacology analysis for potential treatment mechanism of QBW on RSA with PTS

Compounds and therapeutic targets of each TCM ingredient were retrieved from TCMSP with conditions of OB ≥ 30% and DL ≥ 0.18. Pathological targets of PTS (“prothrombotic status”, “thrombophilia”) and RSA (“recurrent pregnancy loss”, “recurrent spontaneous abortion”) were retrieved from GeneCards and OMIM databases, and intersected with QBW targets using InteractiVenn. The ingredient-compound-target network was constructed using Cytoscape 3.10.1. The protein-protein interaction network and the Gene Ontology (GO) enrichment, including Biological Process (BP), Cellular Component (CC) and Molecular Function (MF), and Reactome pathways enrichment were performed on STRING. The top 20 genes and top 10 compounds, ranked by interaction degree, were extracted from each network.

### 2.10 Network toxicology analysis for potential safety issues in QBW in the liver, endometrium and placenta

Compounds of each TCM ingredient were extracted from TCMSP as in 2.9. After all compound names were converted to MeSH IDs, the potential toxic targets of compounds were predicted using the CTD database. The toxic targets were analyzed for tissue-specific enrichment with TissueEnrich,^22^ and the enriched targets specific to the tissue of interest were extracted as the potential toxic targets of QBW on that tissue. The keywords of “hepatotoxicity” and “reproductive toxicity” were used to search GeneCards, OMIM and PharmGKB databases. The subsequent analyses, using Venn diagram, ingredient-compound-target network, protein-protein interaction network, GO and Reactome pathway enrichment, were performed similarly to those in 2.9.

### 2.11 Statistical analysis

All statistical analyses were performed and data were plotted using GraphPad Prism 10 (GraphPad Software, La Jolla, CA, USA). Normality and variance homogeneity were assessed for all endpoints. Variables that passed both were analyzed by one-way ANOVA with Bonferroni post hoc correction, that passed normality but failed variance homogeneity were analyzed using Welch's ANOVA with Dunnett's T3 post hoc correction, and that failed normality were analyzed using the Kruskal-Wallis test with Dunn's post hoc correction. Microcirculation endpoints were analyzed by two-way ANOVA with Bonferroni correction. Confidence intervals were constructed using the pooled error term from the respective ANOVA model. An adjusted *p* < 0.05 was considered statistically significant. All error bars represent 95% confidence intervals, and individual data points are shown in all figures.

## 3 Results

### 3.1 QBW reduced mifepristone-induced miscarriage

The anatomical positions of embryos within the uterine horns are illustrated in Fig. 1A. Rats in the model group exhibited higher miscarriage numbers and rates than controls (mean difference = +5.89 embryos and +88.4%, respectively; both *p* < 0.001; Supplementary Figure S3A, Fig. 1B). QBW at 1.76 and 3.52 g/kg dose-dependently reduced both outcomes (miscarriage number: mean difference = −4.67 and −5.67 embryos, *p* = 0.007 and *p* < 0.001; miscarriage rate: −84.5% and −88.4%, both *p* < 0.001), whereas aspirin had no significant effect (both *p* > 0.99; Supplementary Figure S3A, Fig. 1B).

**Fig. 1:**
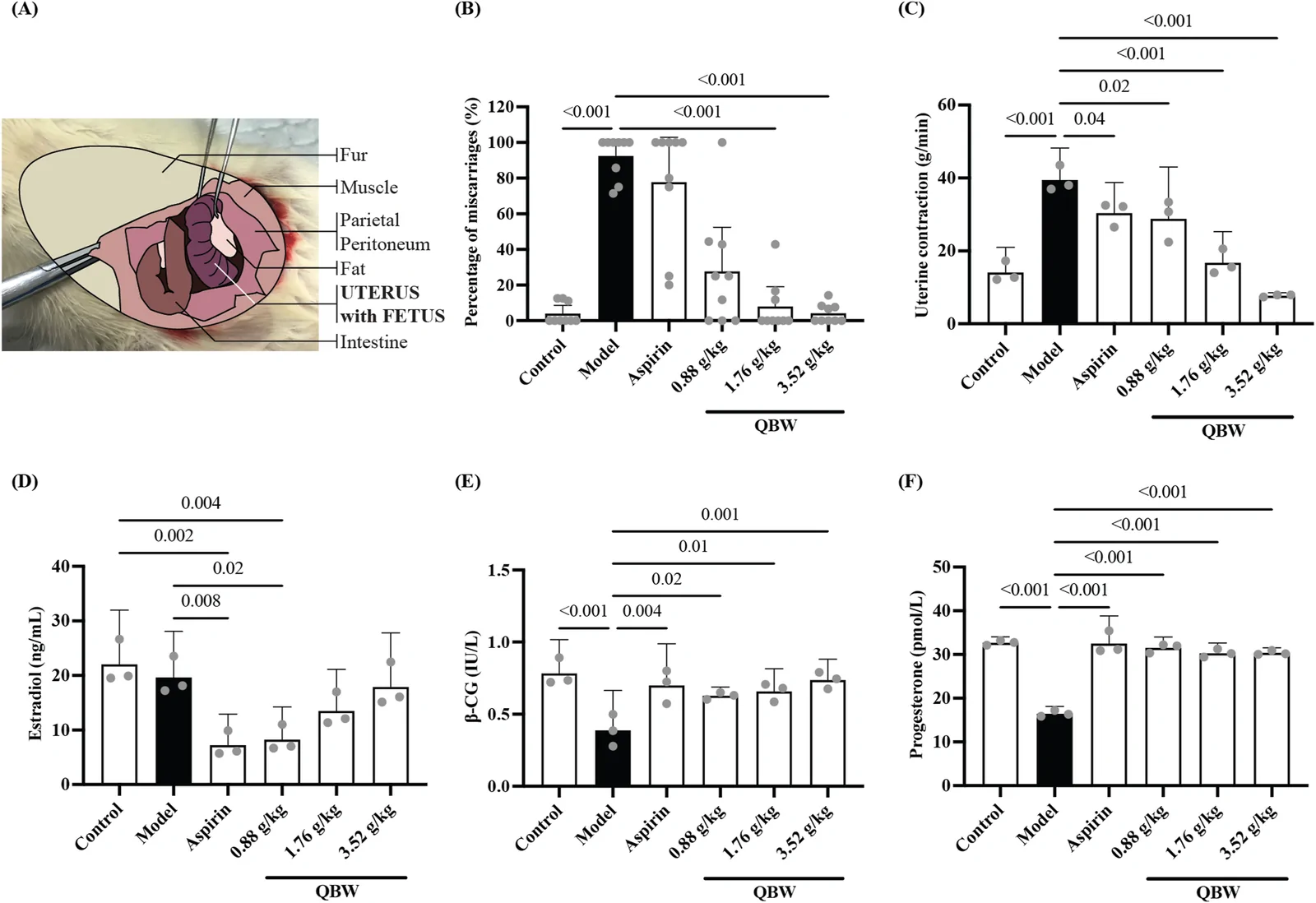
Effect of QBW on mifepristone-induced spontaneous miscarriage in pregnant rats. (A) Schematic of the fetal anatomical position in the uterus; (B) miscarriage rate; (C) uterine contraction intensity; (D) serum estradiol; (E) serum β-CG; (F) serum progesterone. All p-values are shown as numbers, compared with the model group, and with the control group merely for (D). n = 9 for (B), n = 3 for (C-F).

### 3.2 QBW reduced excessive uterine contractility *in vivo* in a rat model of PTS and spontaneous abortion

The model group exhibited a higher mean peak contraction force, contraction frequency and uterine contraction intensity than controls (all *p* < 0.05; Supplementary Figure S3B-C and Fig. 1C). QBW at 1.76 and 3.52 g/kg reduced the peak contraction force and frequency, whereas aspirin had no significant effect (Supplementary Fig. S3B–C). Both aspirin and all doses of QBW reduced the uterine contraction intensity (Fig. 1C). Detailed values are provided in Supplementary Table S1.

### 3.3 QBW reduced miscarriage by improving pregnancy-related hormones

Compared with the control group, β-CG and progesterone levels were halved in the model group (both 0.50-fold; *p* < 0.001), whereas estradiol did not differ significantly (Fig. 1D–F). Aspirin significantly increased β-CG and progesterone to 1.80-fold and 1.97-fold of model levels, but decreased estradiol to 0.37-fold (all *p* < 0.01; Fig. 1D–F). All doses of QBW significantly increased β-CG (to 1.62-fold, 1.70-fold, and 1.90-fold at 0.88, 1.76, and 3.52 g/kg, respectively) and progesterone (to 1.91-fold, 1.84-fold, and 1.84-fold, respectively) compared with the model group (all *p* < 0.05; Fig. 1E and F), while QBW at 0.88 g/kg decreased estradiol to 0.42-fold (*p* = 0.02; Fig. 1D). Compared with the control group, estradiol remained decreased only in the aspirin and QBW 0.88 g/kg groups (0.33-fold and 0.37-fold; *p* = 0.002 and *p* = 0.004; Fig. 1D). Detailed values are provided in Supplementary Table S1.

### 3.4 QBW improved blood stasis in uterine microvessels and capillaries

Representative stereomicroscopic still images of the uterine surface vasculature, captured from the *in vivo* video recordings, are shown in Fig. 2A. Compared with the control group, the model group showed significantly decreased diameter of microvessels and diameter and number of capillaries at 10, 20, and 30 min, whereas number of microvessels did not differ (Fig. 2B–E). Aspirin significantly increased all four parameters, except number of microvessels, at all time points (Fig. 2B–D-E). QBW at 3.52 g/kg increased all four parameters at all time points, whereas QBW at 1.76 g/kg increased diameter of microvessels (at 10 and 30 min) and number of capillaries (at all time points) (Fig. 2B–E). Detailed values are provided in Supplementary Table S2.

**Fig. 2:**
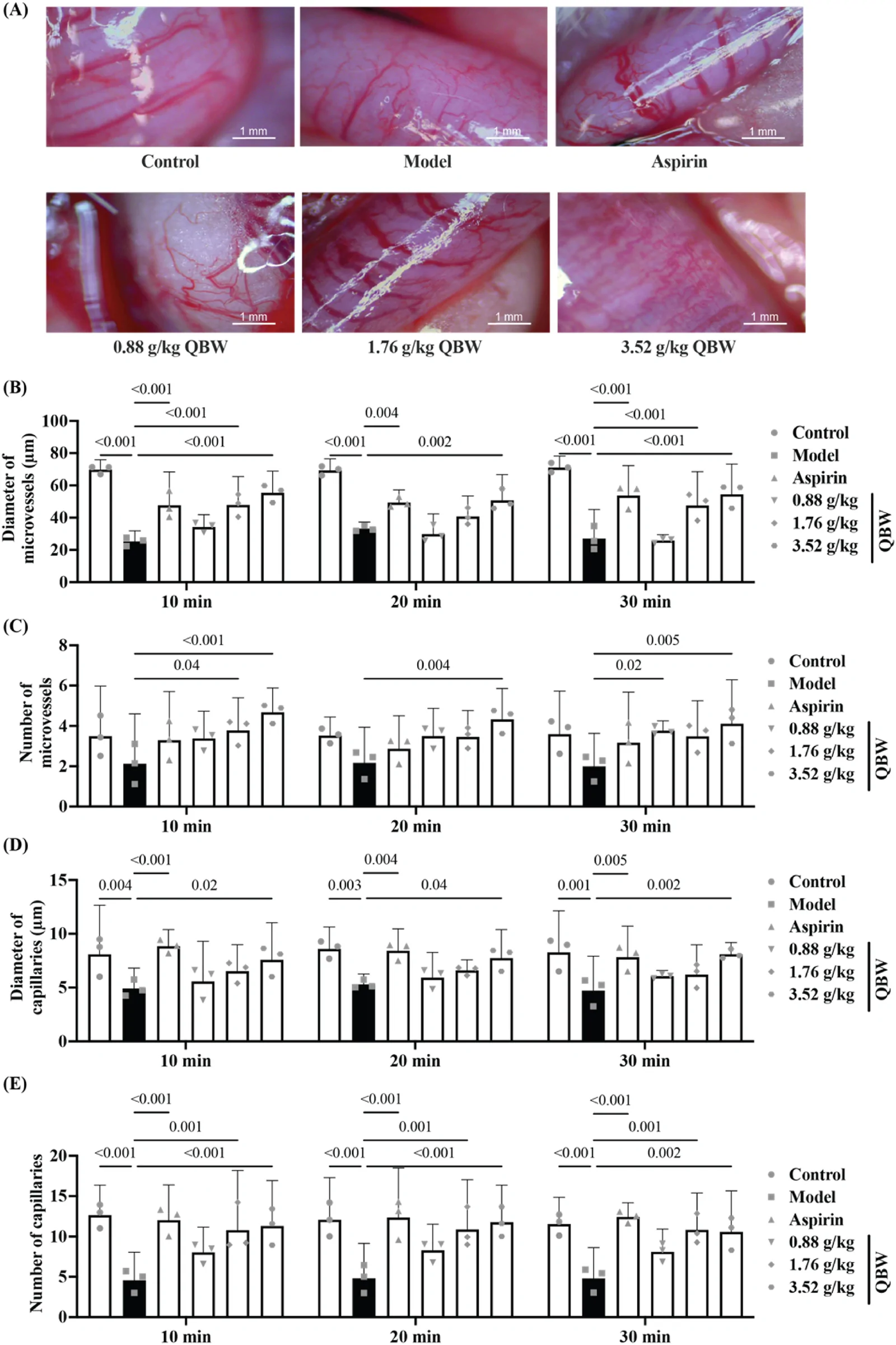
Effect of QBW on uterine microvessels (11-100 μm in diameter) and capillaries (≤10 μm in diameter) in rats with epinephrine-induced PTS. (A) Representative still frames of the uterine surface vasculature from *in vivo* stereomicroscopic recordings in each group; (B) diameter of the microvessels; (C) number of microvessels; (D) diameter of capillaries; (E) number of capillaries. Data were collected at 10, 20, and 30 min after the start of the recording. All p-values are shown as numbers, compared with the model group. n = 3.

### 3.5 QBW prolonged coagulation times and decreased plasma fibrinogen

Compared with the control group, the APTT, PT and TT were shortened in the model group, while fibrinogen was increased (Fig. 3A–D). Aspirin prolonged APTT and TT, and decreased fibrinogen (Fig. 3A–C-D). QBW at all doses also prolonged APTT, PT and TT, and at 1.76 and 3.52 g/kg decreased fibrinogen (Fig. 3A–D). Detailed values are provided in Supplementary Table S3.

**Fig. 3:**
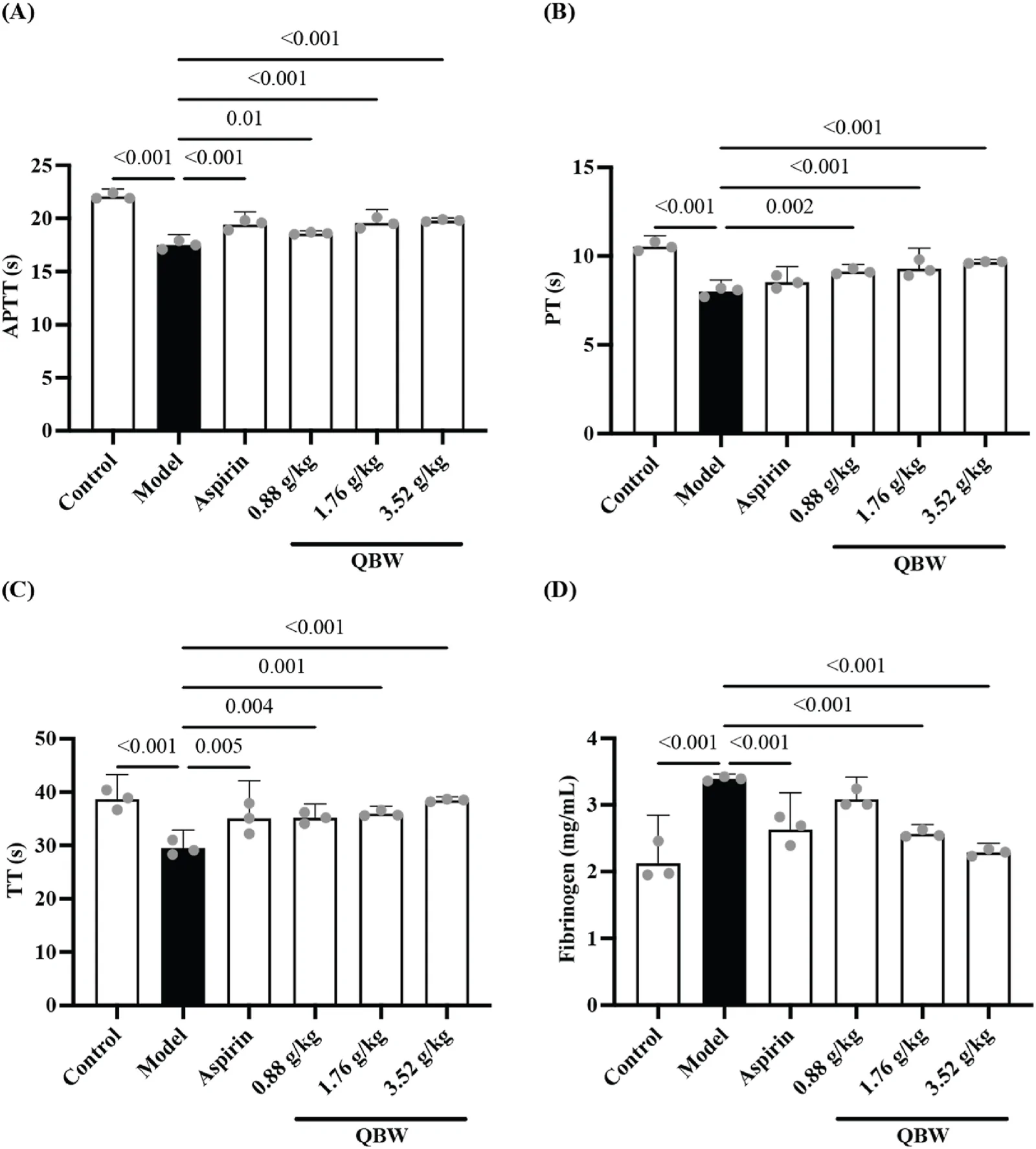
Effect of QBW on coagulation parameters. (A) APTT; (B) PT; (C) TT; (D) plasma concentration of fibrinogen. All p-values are shown as numbers, compared with the model group. n = 3.

### 3.6 QBW reduced thrombosis by restoring the balance between antithrombotic factors and prothrombotic factors

The antithrombotic factors AT-III, 6-keto-PGF_1α_, and prostacyclin were decreased in the model group to 0.76-fold, 0.42-fold, and 0.35-fold of control levels (all *p* ≤ 0.001; Fig. 4A and B and Supplementary Fig. S3D). Aspirin increased them to 1.26-fold, 1.43-fold, and 1.64-fold of model levels (all *p* < 0.05; Fig. 4A and B and Supplementary Fig. S3D). QBW at 3.52 g/kg produced larger increases (AT-III 2.20-fold, 6-keto-PGF_1α_ 1.76-fold, prostacyclin 2.76-fold; all *p* < 0.001), whereas at 0.88 and 1.76 g/kg it selectively increased only AT-III (2.19-fold and 2.54-fold) and prostacyclin (1.53-fold and 2.37-fold) (all *p* < 0.01) (Fig. 4A and B, Supplementary Fig. S3D). Conversely, the prothrombotic factors D-dimer, F1+2, TXA_2_, and TXB_2_ were elevated in the model group to 1.19-fold, 1.44-fold, 1.33-fold, and 1.60-fold (all *p* < 0.001; Fig. 4C–F). Aspirin reduced F1+2, TXA_2_, and TXB_2_ to 0.55-fold, 0.79-fold, and 0.74-fold but had no significant effect on D-dimer (Fig. 4C–F). QBW at 0.88, 1.76, and 3.52 g/kg decreased all four factors: D-dimer to 0.81-fold, 0.78-fold, and 0.88-fold; F1+2 to 0.72-fold, 0.31-fold, and 0.44-fold; TXA_2_ to 0.69-fold, 0.68-fold, and 0.84-fold; and TXB_2_ to 0.89-fold, 0.86-fold, and 0.76-fold (all *p* < 0.05; Fig. 4C–F). Detailed values are provided in Supplementary Table S4.

**Fig. 4:**
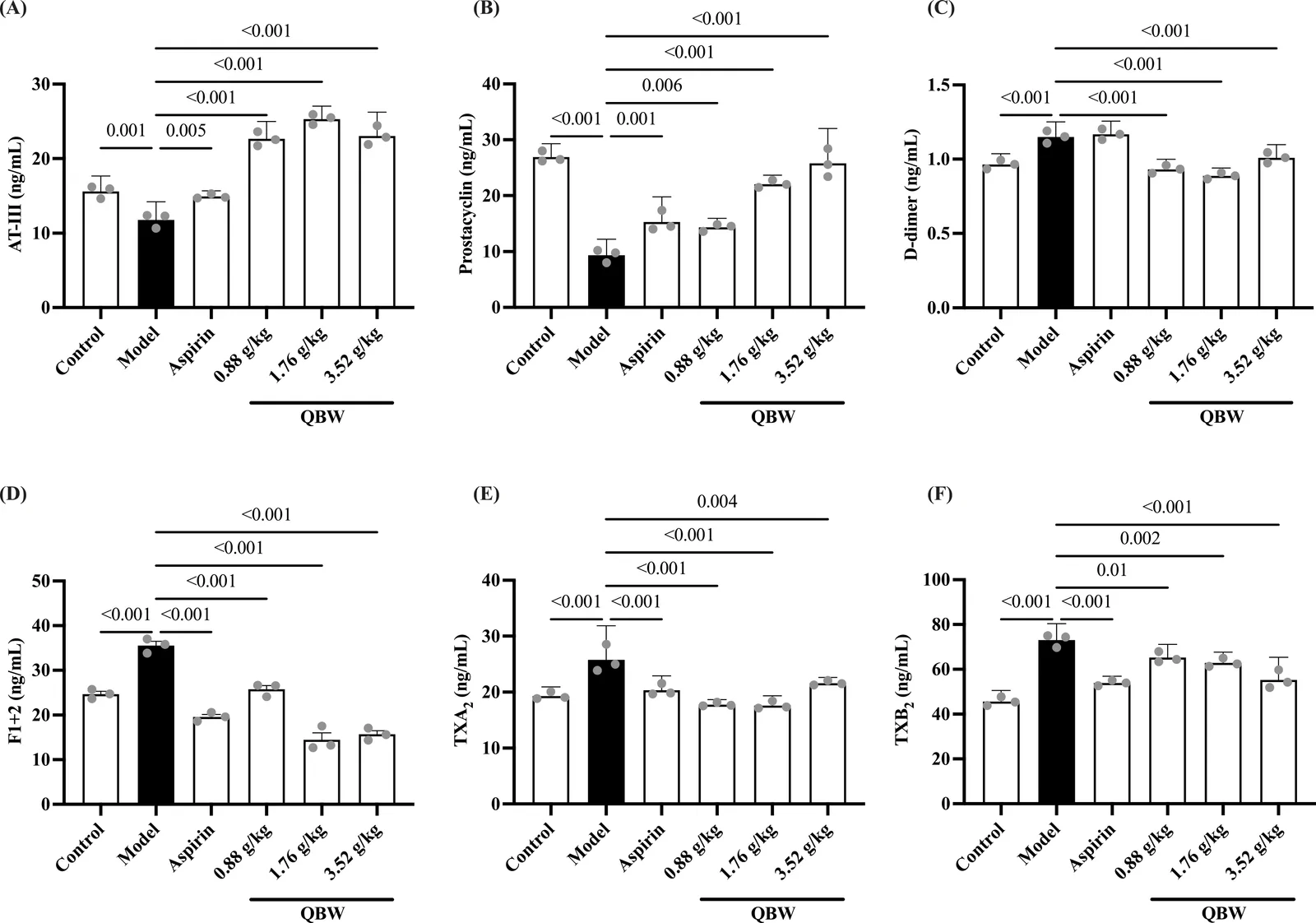
Effect of QBW on the balance between antithrombotic and prothrombotic factors in rats with PTS. (A) Serum AT-III; (B) serum prostacyclin; (C) serum D-dimer; (D) serum F1+2; (E) serum TXA_2_; (F) serum TXB_2_. All p-values are shown as numbers, compared with the model group. n = 3.

### 3.7 Effects of QBW on the relative weights of the uterus, ovaries, kidney, and spleen in pregnant rats

Compared with the control group, the relative weights of the uterus and ovaries were increased, and those of the spleen were decreased in the model group (mean difference = +0.265%, +0.016%, −0.070%, *p* = 0.004, *p* = 0.01, *p* = 0.002) (Fig. 5A–B, D). After QBW treatment, only the 0.88 g/kg group still showed a greater relative uterine weight (mean difference = +0.311%, *p* < 0.001) (Fig. 5A). Other QBW treatment groups did not differ significantly from the control group in any organ (Fig. 5A–D).

**Fig. 5:**
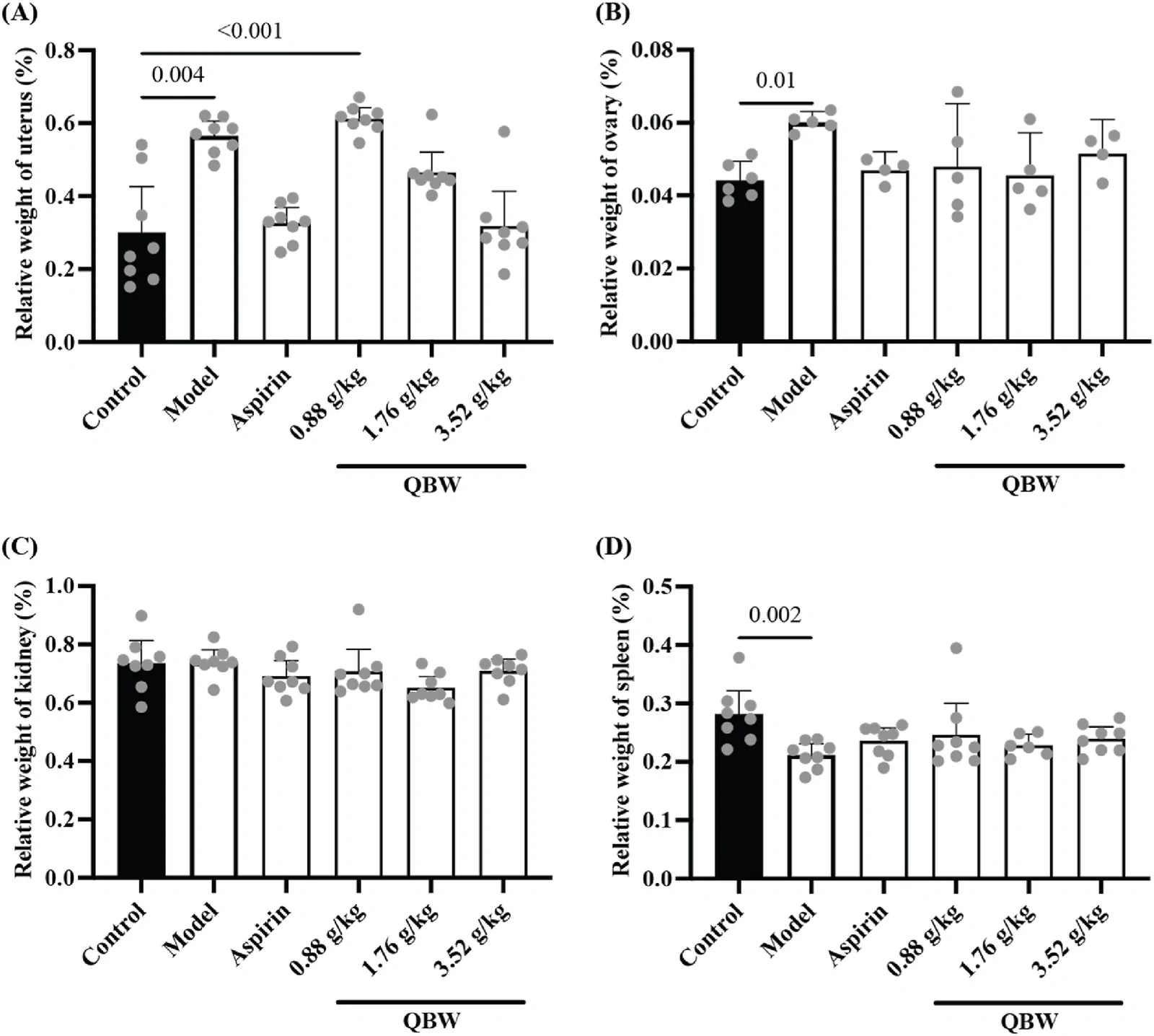
Effect of QBW on the relative weights of the uterus, ovaries, kidney, and spleen in pregnant rats. (A) Relative uterine weight; (B) relative ovarian weight; (C) relative kidney weight; (D) relative spleen weight. All p-values are shown as numbers, compared with the control group. n = 4-8.

### 3.8 QBW may modulate Th2 cytokines to counteract inflammation in RSA with PTS

In total, 229 compounds from the 15 ingredients of QBW and 284 therapeutic targets were retrieved from TCMSP, and 698 and 212 pathological targets of RSA and PTS, respectively, were retrieved from GeneCards and OMIM databases, yielding 29 common targets (Fig. 6A). In the ingredient-compound-target network, the top 5 genes were *PTGS2*, *NOS2*, *F7*, *NOS3*, and *IL6*, and the top 5 compounds were quercetin, β-sitosterol, kaempferol, stigmasterol, and isorhamnetin (Fig. 6B–D). In the STRING protein-protein interaction network, the top 5 genes were *TNF*, *IL6*, *CCL2*, *CXCL8*, and *IFNG* (Fig. 6E and F). The top 5 GO (BP, CC, MF) and Reactome pathway enrichments are shown in Supplementary Fig. S4A–D.

**Fig. 6:**
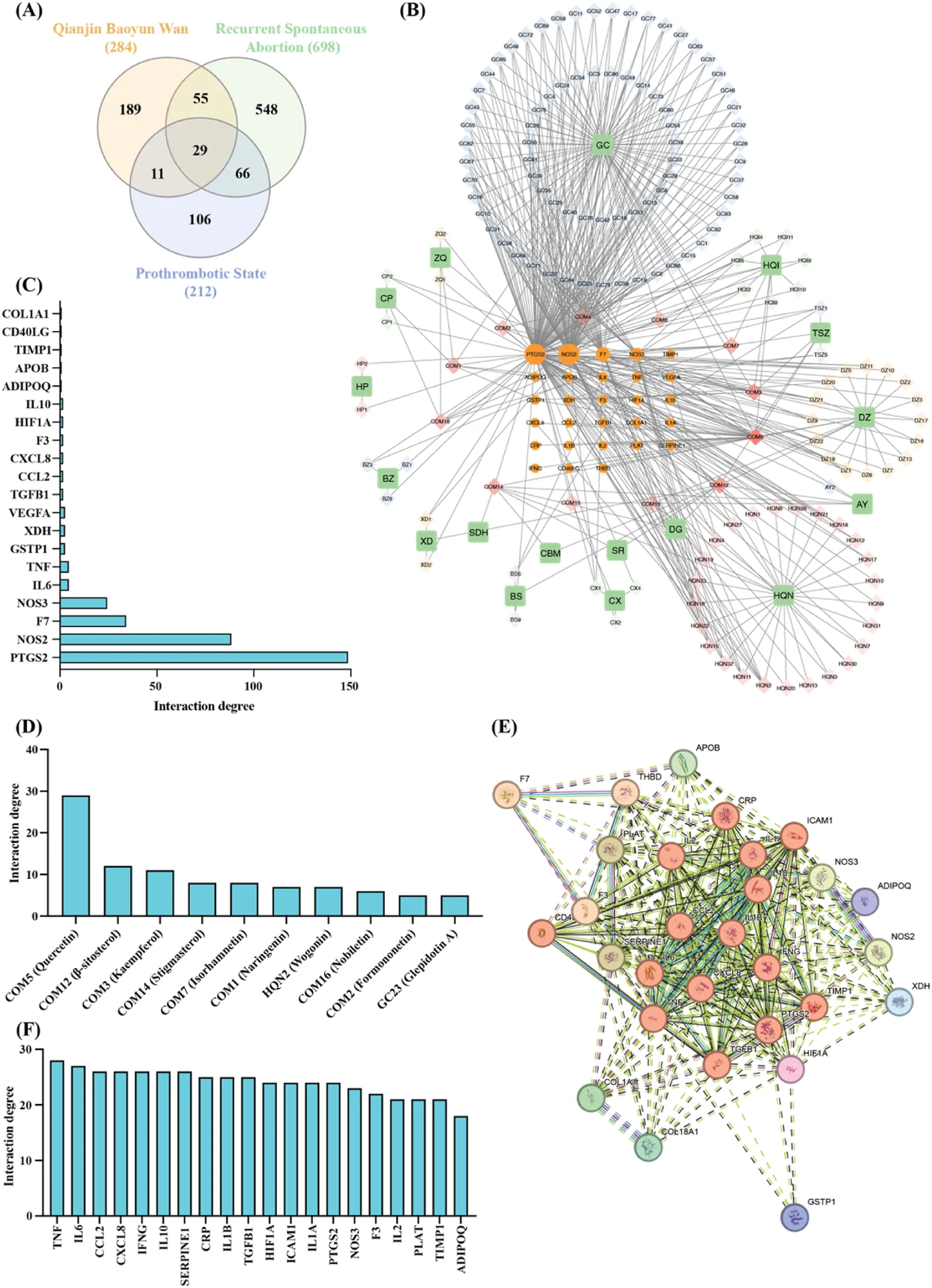
Network pharmacology analysis of QBW against RSA with PTS. (A) Venn diagram of the intersections among QBW therapeutic targets, RSA pathological targets, and PTS pathological targets; (B) ingredient-compound-target network; (C) top 20 genes ranked by degree in the ingredient-compound-target network; (D) top 10 compounds ranked by degree; (E) protein-protein interaction network constructed with STRING; (F) top 20 genes ranked by degree in the protein-protein interaction network.

### 3.9 QBW may disturb hepatic lipid and steroid metabolism

Of 5944 predicted toxicity targets, 204 were enriched in the liver, of which 42 overlapped with hepatotoxicity targets (Supplementary Fig. S5A–C). In the ingredient-compound-target network, the top 5 genes were *CYP3A4*, *UGT1A1*, *UGT2B7*, *UGT1A9*, and *SLCO1B3* (Supplementary Fig. S5D–E). In the protein-protein interaction network, the top 5 genes were *CYP3A4*, *CYP7A1*, *NR1H4*, *APOA1*, and *SULT2A1* (Supplementary Fig. S5F–G). The top 5 GO and Reactome pathway enrichments are shown in Supplementary Fig. S5H–K.

### 3.10 QBW may influence estrogen signaling in the endometrium and placenta

44 and 118 toxicity target genes were enriched in endometrium and placenta, respectively (Fig. 7A and B). One common gene each (*ESR1* for the endometrium and *CYP19A1* for the placenta) was identified between the predicted toxicity targets of QBW and the reproductive toxicity targets (Fig. 7C), and the ingredient-compound-target networks were constructed (Fig. 7D and E).

**Fig. 7:**
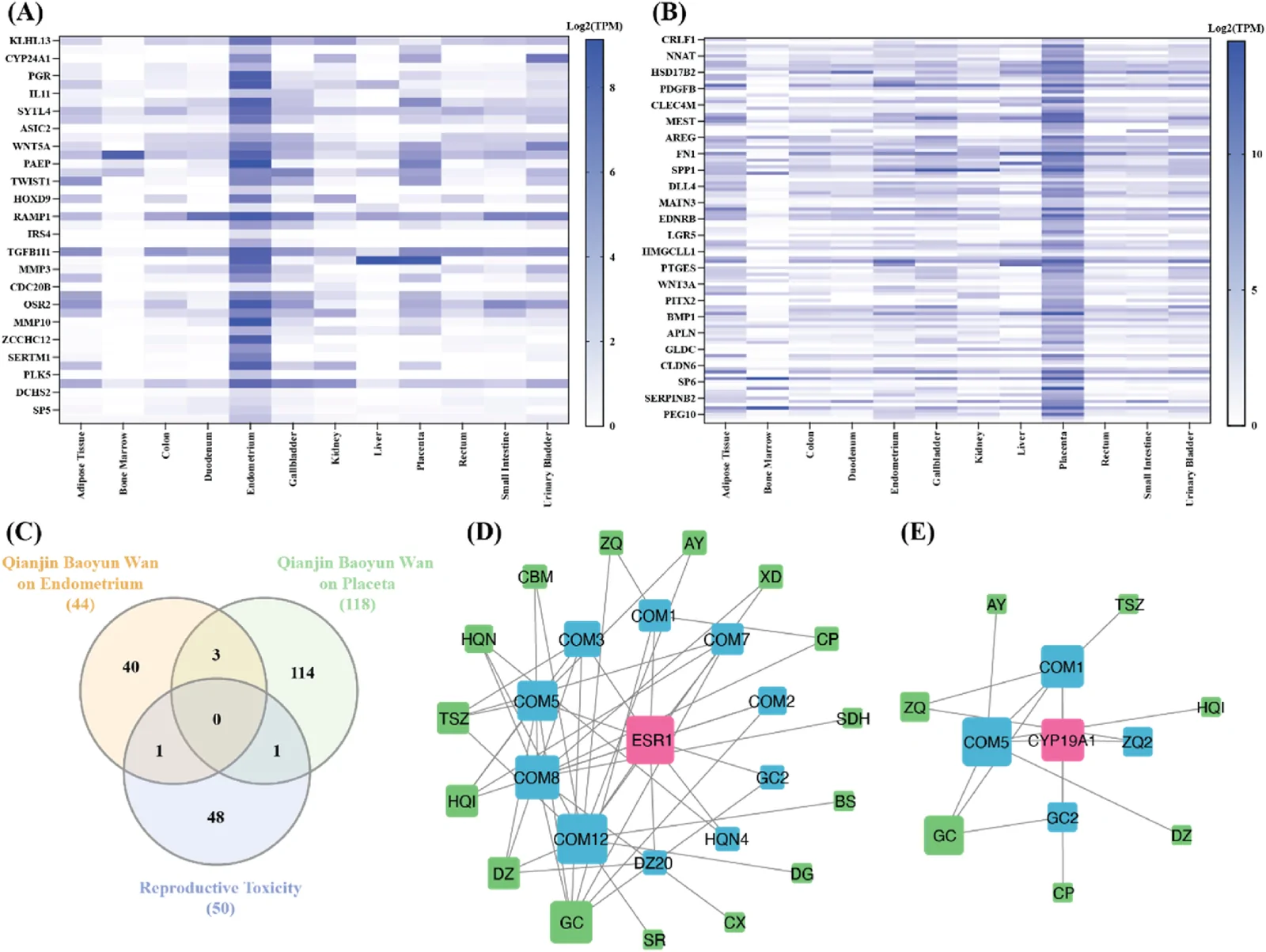
Network toxicology analysis of QBW in the endometrium and placenta. (A) Tissue-specific genes enriched in the endometrium (TissueEnrich); (B) tissue-specific genes enriched in the placenta (TissueEnrich); (C) Venn diagram of the intersections between QBW-predicted toxic targets specific to the endometrium or placenta and reproductive-toxicity targets retrieved from the databases; (D) ingredient-compound-target network of endometrial toxicity; (E) ingredient-compound-target network of placental toxicity.

## 4 Discussion

In this study, we successfully replicated a rat model of RSA with PTS using mifepristone and epinephrine,^21^ and found that QBW reduced miscarriages compared with aspirin. QBW restored estradiol and progesterone, attenuated excessive uterine contractility, improved uterine microcirculation, and rebalanced coagulation parameters and thrombotic factors, whereas aspirin improved only the vascular and coagulation components. Network pharmacology and toxicology analyses further predicted the active compounds, potential mechanisms, and safety profile of QBW, although these predictions require experimental validation.

Differential analysis of the microvasculature sheds light on the vascular pathology of RSA with PTS (the physiological basis of the microvessel-capillary distinction is summarized in Supplementary Discussion S1). QBW, particularly at 3.52 g/kg, restored the diameter and density of both microvessels and capillaries, suggesting improved microvascular perfusion, reduced blood stasis, and restored oxygen and nutrient exchange for the fetus. These findings support the concept that microcirculatory dysfunction is a key downstream consequence of PTS contributing to miscarriage, and that QBW may reduce pregnancy loss by restoring microcirculatory function. Although a direct causal link between microcirculatory restoration and contraction suppression cannot be established in the present study, improved uteroplacental perfusion may indirectly support myometrial quiescence by maintaining the oxygen and nutrient supply to uterine smooth muscle.

Network pharmacology analysis further elucidates the potential mechanisms underlying these observations by identifying key bioactive compounds, including the natural flavonoids quercetin and kaempferol and the phytosterol β-sitosterol, which collectively regulate endocrine homeostasis, vascular and hemostatic balance, and inflammatory responses. Quercetin preserves ovarian aromatase (*CYP19A1*) expression and increases steroidogenic acute regulatory protein (StAR) mRNA expression, providing a plausible mechanistic basis for the estradiol and progesterone recovery observed in QBW-treated rats.^23^ β-Sitosterol primarily exerted indirect endocrine-modulating effects, improving endometrial receptivity and sex hormone balance through estrogen receptor α signaling and modulation of gut microbiota.^24^^,^^25^ Notably, phytosterol accumulation induces reversible infertility in mice lacking ABCG5/ABCG8 transporters,^26^ although this effect is genotype-dependent and is unlikely to reflect physiological conditions in normal pregnancy.

In terms of vascular and hemostatic balance, quercetin improves microcirculatory perfusion by activating endothelial nitric oxide synthase (eNOS) and thereby increasing nitric oxide (NO) production, consistent with the recovery of microvessel and capillary diameters observed in this study.^27^^,^^28^ Kaempferol preserves vascular integrity through activation of the Nrf2/HO-1 pathway.^29^ Both quercetin and kaempferol exert antiplatelet and antithrombotic effects, with quercetin regulating platelet activation via NF-κB signaling,^29^, ^30^, ^31^ and β-sitosterol exerts anticoagulant effects by inhibiting thrombin activity and reducing platelet activation.^32^ In addition, quercetin interacts with epidermal growth factor receptor (EGFR), alleviating placental endothelial dysfunction induced by trophoblast ischemia and mitigating hypoxia-reperfusion injury-associated fetal loss, which may additionally support placental endothelial protection under ischemic conditions.^33^ Collectively, these compounds may contribute to reducing thrombotic risk and maintaining microcirculatory stability.

The attenuation of excessive uterine contractility may also be partially explained by the anti-inflammatory properties of QBW's active compounds: quercetin and kaempferol reduce pro-inflammatory cytokines and suppress NF-κB/PTGS2-mediated prostaglandin production,^31^^,^^34^ and enrichment analysis identified IL-10 and IL-4/IL-13 signaling among the pathways targeted by QBW (Supplementary Fig. S4), suggesting a potential shift toward an anti-inflammatory immune state favorable for pregnancy maintenance. Consistent with the hierarchical interpretation proposed below, these anti-inflammatory and immunomodulatory effects are more likely to represent complementary rather than primary mechanisms, and all network-derived mechanisms should be interpreted as hypothesis-generating (Supplementary Discussion S2). The mechanism summary of the three major compounds is shown in Fig. 8.

**Fig. 8:**
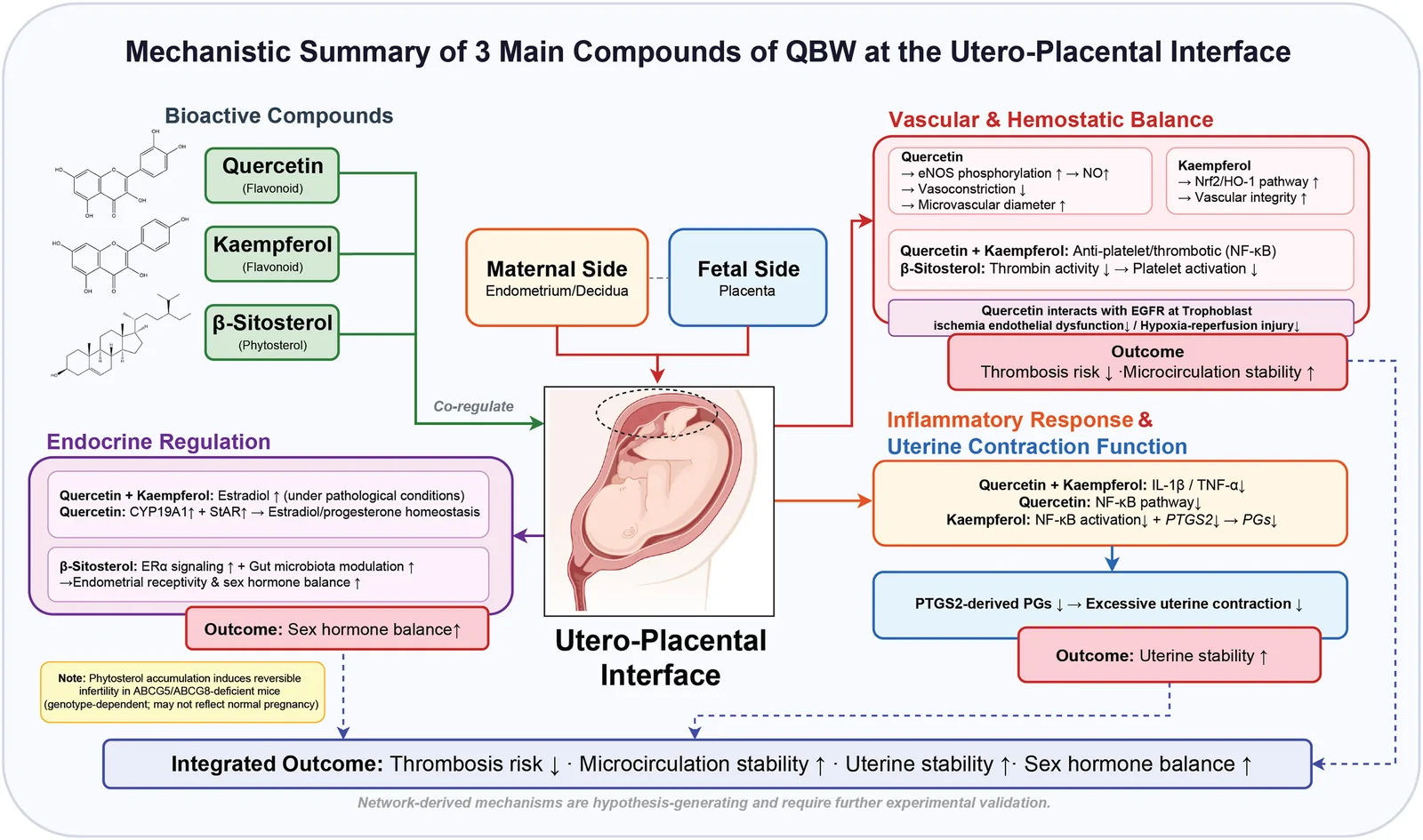
The mechanistic summary of quercetin, kaempferol and β-sitosterol at the uteroplacental interface managing RSA with PTS.

Clinically, LDA-related antiplatelet and LMWH-related anticoagulant therapies are widely used in RSA patients with PTS or suspected thrombophilia, yet their capabilities of improving live births remain inconsistent.^15^^,^^16^ In our study, aspirin partially improved coagulation and uterine microcirculation but failed to improve miscarriage induced by progesterone withdrawal, which is consistent with these clinical observations. Notably, the aspirin dose used here (6.75 mg/kg in rats, human equivalent ∼75 mg/day) falls within the low-dose range recommended by NICE, WHO, and ACCP (Supplementary Discussion S3), and its limited efficacy likely reflects the non-thrombotic pathological drive of the model rather than a failure of antiplatelet therapy per se. Its partial responsiveness nonetheless validates the PTS component of the model. QBW is derived from XGJAD (Jiao Ai Decoction), preserving the core ingredient composition despite the change in dosage form; studies on XGJAD have demonstrated both clinical efficacy in threatened abortion and pro-angiogenic effects through VEGF signaling, suggesting that QBW may share these effects.^19^^,^^35^ Collectively, our findings provide laboratory evidence that QBW not only exerts an antithrombotic effect comparable to that of aspirin but also improves pregnancy outcomes, suggesting that multi-target modulation by XGJAD-based TCM formulae may be superior to anticoagulation alone for RSA patients with PTS.

Hormonal regulation is central to pregnancy maintenance. Progesterone withdrawal induced by mifepristone in the model mimics luteal insufficiency, leading to uterine instability and fetal loss. Notably, both aspirin and QBW increased progesterone in the present model, suggesting that improved uterine microcirculation may partially preserve gestational endocrine function following mifepristone-induced progesterone withdrawal.^36^ However, their effects on estradiol diverged remarkably. Aspirin and low-dose QBW decreased estradiol, which may reflect inhibited aromatase activity rather than a compensatory adaptation, although off-target effects cannot be excluded.^37^ In contrast, higher-dose QBW restored estradiol together with progesterone, consistent with *CYP19A1* and StAR function, as discussed above.

Estradiol upregulates uterine progesterone receptor (PR) expression and maintains PR signaling efficiency, and progesterone promotes uterine quiescence via the PR.^38^ When PRs are competitively occupied by mifepristone, maintaining progesterone without preserving estradiol to support PR expression may be insufficient to fully restore quiescence signaling, explaining the incomplete contraction suppression by aspirin despite its progesterone-maintaining effect. The dual restoration of both hormones by QBW therefore suggests an endocrine-protective mechanism that extends beyond vascular support, preserving ovarian and placental steroidogenesis to re-establish hormonal homeostasis.^39^ Together with the finding that aspirin maintained progesterone yet achieved only partial contraction suppression, whereas QBW preserved both hormones and suppressed all contraction parameters, these results provide the experimental basis for a hierarchical model in which endocrine stabilization serves as the most upstream action of QBW, providing the hormonal foundation for myometrial quiescence, with the restoration of uterine microcirculation and coagulation balance representing downstream vascular effects.

Beyond efficacy, the safety profile of QBW is important for translational consideration. The changes in the relative weights of the uterus, ovaries, and spleen in the model group implicate pathological processes in RSA-associated uterine contractility, endocrine disturbance, and systemic immune stress. In QBW-treated groups, the relative organ weights did not differ from those of the control group, except for a greater relative uterine weight at 0.88 g/kg, indicating that no adverse organ effects were detected at the doses tested. The tendency toward lower uterine coefficients in QBW-treated groups may reflect the alleviation of thrombotic edema and impaired perfusion, consistent with the microcirculatory improvements described above. In addition, network toxicology analysis flagged hepatic targets clustering around xenobiotic metabolism and steroid turnover (Supplementary Fig. S5). Given the absence of observable hepatic toxicity (unchanged relative organ weights and mortality), this signal is interpreted as increased hepatic metabolic engagement rather than direct injury (Supplementary Discussion S4).

The reproductive safety profile reflects a dual therapeutic-risk window centered on estrogen-regulatory biology. *ESR1* and *CYP19A1* emerged as predicted concern nodes, yet these same pathways overlap with QBW's endocrine rescue mechanism, indicating shared sensitivity between efficacy and liability. Importantly, their biological direction is tissue-stage-dependent and gestational-stage-dependent. Endometrial estrogen receptor alpha suppression favors progesterone-dominant receptivity during implantation, whereas maintained or moderately increased placental *CYP19A1* supports estradiol synthesis and trophoblast function (Supplementary Discussion S5). Collectively, these findings suggest that the principal reproductive safety consideration lies in dose optimization within a gestational-stage-specific window, rather than intrinsic reproductive or hepatic toxicity, although the network-derived predictions require further validation.

## 5 Limitations

Several limitations should be acknowledged. First, the scope of the model should be defined: it combines mifepristone-induced progesterone withdrawal, which drives uterine contractility and pregnancy loss, with epinephrine-induced sympathetic activation, which contributes to coagulation imbalance and microcirculatory dysfunction, and QBW appeared to act on both components (Supplementary Fig. S6). The model therefore represents acute, multifactorial pregnancy destabilization rather than chronic immune-mediated RSA, and is best suited for studying endocrine insufficiency combined with acquired prothrombotic states (a detailed comparison with classical stress-induced pregnancy-loss models is provided in Supplementary Discussion S6).^11^^,^^40^ Future single-hit comparator studies would help clarify the specific contribution of each pathogenic component. Second, histopathological evaluation of the uterus, placenta, and liver, as well as long-term reproductive safety studies, were beyond the scope of this study and should be prioritized in future investigations. Third, although microcirculation images were independently analyzed by two blinded observers, the manual midline method is susceptible to sampling variability, and physiological factors, such as respiratory motion, uterine peristalsis, and anesthetic depth, may have contributed to measurement error. Inter-observer agreement was not formally assessed, and automated quantification software is recommended for future studies to improve reproducibility. Fourth, the biological relevance of serum β-CG in rats remains uncertain, and hormone detection by ELISA should be cross-validated by LC-MS or RIA in future work.^41^ Fifth, the molecular mechanisms proposed from network pharmacology and toxicology remain hypothesis-generating and require direct experimental validation, and alternative enrichment tools such as KEGG and WikiPathway may provide different results. Lastly, the small sample size (n = 3) for many secondary outcomes limits the precision of effect estimates and increases the risk of both Type I and Type II errors.

## 6 Conclusion

In conclusion, QBW reduced miscarriage and excessive uterine contractility and improved pregnancy-related hormones in a dual-hit rat model of RSA with PTS, while also restoring uterine microcirculation, prolonging coagulation time, and rebalancing antithrombotic and prothrombotic factors. Network pharmacology and toxicology analyses further predicted the potential active compounds and their pharmacological and toxicological mechanisms, suggesting that QBW may have therapeutic potential for RSA patients with PTS. Future studies should validate the mechanisms by which QBW restores estradiol, characterize the pharmacokinetic profiles of its active compounds, and explore dose optimization strategies. Immune profiling at the maternal-fetal interface, placental histopathology, and validation in additional RSA models would further strengthen translational relevance, and controlled clinical studies in carefully stratified RSA populations are ultimately warranted to confirm these preclinical findings.

## Ethics approval and consent to participate

All experimental procedures and animal welfare protocols strictly followed the National Research Council's Guide for the Care and Use of Laboratory Animals and were ethically approved by the Animal Care and Use Committee of the Institute of Biological and Medical Engineering, Guangdong Academy of Sciences (No. K2022-01-022). The pregnant animal welfare was strictly discussed and ensured before ethical approval.

## Availability of data and materials

The datasets used and/or analyzed during the current study are available from the corresponding author on reasonable request.

## Authors' contributions

R.Q. and H.N. conceptualized, supervised and administered the study; H.N., R.Q., H.Z., T.W. and MY.Y. designed the methodology for research; H.Z., T.W., MQ.Y. and A.M. finished the investigation; H.Z., T.W., MQ.Y. and H.D. finished the formal analysis; A.M., J.P., Q.D. and MY.Y. validated the results; H.Z. and H.D. prepared all visuals and figures; H.Z., A.M. and H.D. drafted the original manuscript, and all authors reviewed & edited the manuscript.

## Funding

This work was supported by grants from the National Natural Science Foundation of China to H.N. (No. 81373993), China Postdoctoral Science Foundation to Q.D. (No. 2023M740850), and Science and Technology Project in Guangzhou (No. 202102070001).

## Declaration of competing interest

The authors declare that they have no known competing financial interests or personal relationships that could have appeared to influence the work reported in this paper.
