# Supplementary material for: Qianjin Baoyun Wan reduces spontaneous abortion and the prothrombotic state in pregnant rats
Source: J Tradit Complement Med. 2026 Sep 23;16(6):678–89. doi: 10.1016/j.jtcme.2026.09.006 (PMC13729029; doi:10.1016/j.jtcme.2026.09.006)
Supplement: Supplementary file 1 [file mmc1.docx]

# Supplementary Materials

Supplementary Table S1. Effect estimates and 95% confidence intervals for uterine contraction and hormone outcomes (n=3).

| Outcome | Comparison | Mean Diff. | 95% CI  (Lower, Upper) | p |
| --- | --- | --- | --- | --- |
| *Uterine contraction* |  |  |  |  |
| Peak contraction force (g) | Model vs. Control | +0.217 | (0.0297, 0.404) | 0.02 |
|  | QBW 1.76 g/kg vs. Model | -0.393 | (-0.580, -0.206) | <0.001 |
|  | QBW 3.52 g/kg vs. Model | -0.563 | (-0.750, -0.376) | <0.001 |
| Contraction frequency (/min) | Model vs. Control | +25.3 | (22.2, 28.4) | <0.001 |
|  | QBW 1.76 g/kg vs. Model | -8.33 | (-11.4, -5.22) | <0.001 |
|  | QBW 3.52 g/kg vs. Model | -16.7 | (-19.8, -13.6) | <0.001 |
| Uterine contraction intensity (g/min) | Model vs. Control | +25.4 | (16.6, 34.3) | <0.001 |
|  | Aspirin vs. Model | -9.10 | (-18.0, -0.232) | 0.04 |
|  | QBW 0.88 g/kg vs. Model | -10.6 | (-19.5, -1.78) | 0.02 |
|  | QBW 1.76 g/kg vs. Model | -22.8 | (-31.6, -13.9) | <0.001 |
|  | QBW 3.52 g/kg vs. Model | -31.8 | (-40.6, -22.9) | <0.001 |
| *Hormones* |  |  |  |  |
| Estradiol (ng/mL) | Aspirin vs. Model | -12.4 | (-20.6, -4.25) | 0.008 |
|  | QBW 0.88 g/kg vs. Model | -11.4 | (-19.5, -3.24) | 0.02 |
|  | Aspirin vs. Control | -14.8 | (-24.5, -5.06) | 0.002 |
|  | QBW 0.88 g/kg vs. Control | -13.8 | (-23.5, -4.05) | 0.004 |
| β-CG (IU/L) | Model vs. Control | -0.395 | (-0.606, -0.185) | <0.001 |
|  | Aspirin vs. Model | +0.312 | (0.101, 0.522) | 0.004 |
|  | QBW 0.88 g/kg vs. Model | +0.240 | (0.0296, 0.451) | 0.02 |
|  | QBW 1.76 g/kg vs. Model | +0.270 | (0.0597, 0.481) | 0.01 |
|  | QBW 3.52 g/kg vs. Model | +0.350 | (0.139, 0.560) | 0.001 |
| Progesterone (pmol/L) | Model vs. Control | -16.2 | (-19.3, -13.1) | <0.001 |
|  | Aspirin vs. Model | +16.0 | (12.9, 19.1) | <0.001 |
|  | QBW 0.88 g/kg vs. Model | +15.0 | (11.9, 18.1) | <0.001 |
|  | QBW 1.76 g/kg vs. Model | +13.8 | (10.7, 16.9) | <0.001 |
|  | QBW 3.52 g/kg vs. Model | +14.0 | (10.8, 17.0) | <0.001 |

Abbreviations: CI, confidence interval; Diff., difference. Estradiol did not differ significantly between Model and Control; therefore, comparisons vs. Control are shown here. All comparisons are against the model group unless stated otherwise.

Supplementary Table S2. Effect estimates and 95% confidence intervals for microcirculation outcomes (n=3).

| Outcome | Time | Comparison | Mean Diff. | 95% CI (Lower, Upper) | p |
| --- | --- | --- | --- | --- | --- |
| Diameter of microvessels (μm) | 10 min | Model vs. Control | -44.5 | (-56.6, -32.5) | <0.001 |
|  |  | Aspirin vs. Model | +22.4 | (10.3, 34.4) | <0.001 |
|  |  | QBW 1.76 g/kg vs. Model | +22.7 | (10.6, 34.7) | <0.001 |
|  |  | QBW 3.52 g/kg vs. Model | +30.1 | (18.1, 42.1) | <0.001 |
|  | 20 min | Model vs. Control | -36.2 | (-48.3, -24.2) | <0.001 |
|  |  | Aspirin vs. Model | +16.3 | (4.21, 28.3) | 0.004 |
|  |  | QBW 3.52 g/kg vs. Model | +17.6 | (5.55, 29.6) | 0.002 |
|  | 30 min | Model vs. Control | -44.1 | (-56.1, -32.0) | <0.001 |
|  |  | Aspirin vs. Model | +26.8 | (14.7, 38.8) | <0.001 |
|  |  | QBW 1.76 g/kg vs. Model | +20.6 | (8.60, 32.7) | <0.001 |
|  |  | QBW 3.52 g/kg vs. Model | +27.5 | (15.5, 39.5) | <0.001 |
| Number of microvessels | 10 min | QBW 1.76 g/kg vs. Model | +1.65 | (0.0470, 3.26) | 0.04 |
|  |  | QBW 3.52 g/kg vs. Model | +2.55 | (0.944, 4.16) | <0.001 |
|  | 20 min | QBW 3.52 g/kg vs. Model | +2.16 | (0.550, 3.76) | 0.004 |
|  | 30 min | QBW 0.88 g/kg vs. Model | +1.77 | (0.164, 3.38) | 0.02 |
|  |  | QBW 3.52 g/kg vs. Model | +2.11 | (0.507, 3.72) | 0.005 |
| Diameter of capillaries (μm) | 10 min | Model vs. Control | -3.18 | (-5.52, -0.830) | 0.004 |
|  |  | Aspirin vs. Model | +3.92 | (1.58, 6.27) | <0.001 |
|  |  | QBW 3.52 g/kg vs. Model | +2.67 | (0.323, 5.02) | 0.02 |
|  | 20 min | Model vs. Control | -3.30 | (-5.65, -0.953) | 0.003 |
|  |  | Aspirin vs. Model | +3.14 | (0.793, 5.49) | 0.004 |
|  |  | QBW 3.52 g/kg vs. Model | +2.45 | (0.0995, 4.79) | 0.04 |
|  | 30 min | Model vs. Control | -3.56 | (-5.91, -1.22) | 0.001 |
|  |  | Aspirin vs. Model | +3.10 | (0.753, 5.45) | 0.005 |
|  |  | QBW 3.52 g/kg vs. Model | +3.39 | (1.04, 5.73) | 0.002 |
| Number of capillaries | 10 min | Model vs. Control | -8.07 | (-12.2, -3.98) | <0.001 |
|  |  | Aspirin vs. Model | +7.45 | (3.36, 11.5) | <0.001 |
|  |  | QBW 1.76 g/kg vs. Model | +6.22 | (2.13, 10.3) | 0.001 |
|  |  | QBW 3.52 g/kg vs. Model | +6.74 | (2.65, 10.8) | <0.001 |
|  | 20 min | Model vs. Control | -7.27 | (-11.4, -3.18) | <0.001 |
|  |  | Aspirin vs. Model | +7.54 | (3.45, 11.6) | <0.001 |
|  |  | QBW 1.76 g/kg vs. Model | +6.06 | (1.97, 10.1) | 0.001 |
|  |  | QBW 3.52 g/kg vs. Model | +6.96 | (2.87, 11.0) | <0.001 |
|  | 30 min | Model vs. Control | -6.76 | (-10.8, -2.67) | <0.001 |
|  |  | Aspirin vs. Model | +7.63 | (3.54, 11.7) | <0.001 |
|  |  | QBW 1.76 g/kg vs. Model | +6.04 | (1.95, 10.1) | 0.001 |
|  |  | QBW 3.52 g/kg vs. Model | +5.77 | (1.68, 9.86) | 0.002 |

CI, confidence interval; Diff., difference. All comparisons are against the model group unless stated otherwise.

Supplementary Table S3. Effect estimates and 95% confidence intervals for coagulation parameters (n=3).

| Outcome | Comparison | Mean Diff. | 95% CI  (Lower, Upper) | p |
| --- | --- | --- | --- | --- |
| APTT (s) | Model vs. Control | -4.57 | (-5.44, -3.69) | <0.001 |
|  | Aspirin vs. Model | +1.93 | (1.06, 2.81) | <0.001 |
|  | QBW 0.88 g/kg vs. Model | +1.10 | (0.224, 1.98) | 0.01 |
|  | QBW 1.76 g/kg vs. Model | +2.07 | (1.19, 2.94) | <0.001 |
|  | QBW 3.52 g/kg vs. Model | +2.30 | (1.42, 3.18) | <0.001 |
| PT (s) | Model vs. Control | -2.53 | (-3.25, -1.82) | <0.001 |
|  | QBW 0.88 g/kg vs. Model | +1.13 | (0.418, 1.85) | 0.002 |
|  | QBW 1.76 g/kg vs. Model | +1.30 | (0.585, 2.02) | <0.001 |
|  | QBW 3.52 g/kg vs. Model | +1.67 | (0.952, 2.38) | <0.001 |
| TT (s) | Model vs. Control | -9.20 | (-13.1, -5.26) | <0.001 |
|  | Aspirin vs. Model | +5.60 | (1.66, 9.54) | 0.005 |
|  | QBW 0.88 g/kg vs. Model | +5.70 | (1.76, 9.64) | 0.004 |
|  | QBW 1.76 g/kg vs. Model | +6.50 | (2.56, 10.4) | 0.001 |
|  | QBW 3.52 g/kg vs. Model | +9.00 | (5.06, 12.9) | <0.001 |
| Fibrinogen (mg/mL) | Model vs. Control | +1.26 | (0.860, 1.67) | <0.001 |
|  | Aspirin vs. Model | -0.757 | (-1.16, -0.354) | <0.001 |
|  | QBW 1.76 g/kg vs. Model | -0.823 | (-1.23, -0.420) | <0.001 |
|  | QBW 3.52 g/kg vs. Model | -1.10 | (-1.51, -0.700) | <0.001 |

CI, confidence interval; Diff., difference. All comparisons are against the model group unless stated otherwise.

Supplementary Table S4. Effect estimates and 95% confidence intervals for thrombosis factors (n=3).

| Outcome | Comparison | Mean Diff. | 95% CI  (Lower, Upper) | p |
| --- | --- | --- | --- | --- |
| *Antithrombotic factors* |  |  |  |  |
| AT-III (ng/mL) | Model vs. Control | -3.81 | (-6.03, -1.60) | 0.001 |
|  | Aspirin vs. Model | +3.14 | (0.924, 5.36) | 0.005 |
|  | QBW 0.88 g/kg vs. Model | +10.9 | (8.64, 13.1) | <0.001 |
|  | QBW 1.76 g/kg vs. Model | +13.5 | (11.3, 15.8) | <0.001 |
|  | QBW 3.52 g/kg vs. Model | +11.3 | (9.06, 13.5) | <0.001 |
| 6-keto-PGF_1α_ (ng/mL) | Model vs. Control | -12.9 | (-16.6, -9.20) | <0.001 |
|  | Aspirin vs. Model | +3.97 | (-7.66, -0.277) | 0.03 |
|  | QBW 3.52 g/kg vs. Model | +7.00 | (-10.7, -3.31) | <0.001 |
| Prostacyclin (ng/mL) | Model vs. Control | -17.6 | (-21.2, -14.0) | <0.001 |
|  | Aspirin vs. Model | +5.99 | (-9.61, -2.37) | 0.001 |
|  | QBW 0.88 g/kg vs. Model | +5.01 | (-8.63, -1.39) | 0.006 |
|  | QBW 1.76 g/kg vs. Model | +12.8 | (-16.4, -9.13) | <0.001 |
|  | QBW 3.52 g/kg vs. Model | +16.5 | (-20.1, -12.9) | <0.001 |
| *Prothrombotic factors* |  |  |  |  |
| D-dimer (ng/mL) | Model vs. Control | +0.186 | (0.105, 0.266) | <0.001 |
|  | QBW 0.88 g/kg vs. Model | -0.218 | (0.137, 0.298) | <0.001 |
|  | QBW 1.76 g/kg vs. Model | -0.262 | (0.182, 0.343) | <0.001 |
|  | QBW 3.52 g/kg vs. Model | -0.14 | (0.0598, 0.221) | <0.001 |
| F1+2 (ng/mL) | Model vs. Control | +10.9 | (6.84, 14.9) | <0.001 |
|  | Aspirin vs. Model | -16.0 | (12.0, 20.0) | <0.001 |
|  | QBW 0.88 g/kg vs. Model | -9.80 | (5.79, 13.8) | <0.001 |
|  | QBW 1.76 g/kg vs. Model | -21.1 | (17.1, 25.1) | <0.001 |
|  | QBW 3.52 g/kg vs. Model | -19.9 | (15.9, 23.9) | <0.001 |
| TXA_2_ (ng/mL) | Model vs. Control | +6.46 | (3.54, 9.38) | <0.001 |
|  | Aspirin vs. Model | -5.43 | (2.52, 8.35) | <0.001 |
|  | QBW 0.88 g/kg vs. Model | -8.01 | (5.09, 10.9) | <0.001 |
|  | QBW 1.76 g/kg vs. Model | -8.16 | (5.24, 11.1) | <0.001 |
|  | QBW 3.52 g/kg vs. Model | -4.18 | (1.26, 7.09) | 0.004 |
| TXB_2_ (ng/mL) | Model vs. Control | +27.4 | (21.0, 33.8) | <0.001 |
|  | Aspirin vs. Model | -19.2 | (12.8, 25.6) | <0.001 |
|  | QBW 0.88 g/kg vs. Model | -7.81 | (1.41, 14.2) | 0.01 |
|  | QBW 1.76 g/kg vs. Model | -10.1 | (3.74, 16.5) | 0.002 |
|  | QBW 3.52 g/kg vs. Model | -17.8 | (11.4, 24.2) | <0.001 |

CI, confidence interval; Diff., difference. All comparisons are against the model group unless stated otherwise.

Supplementary Figure S1. The modeling and drug administration procedure

Supplementary Figure S2. Method for counting and measuring microvessels and capillaries in perimetrial images. The midline along the perimetrium is marked in yellow. The 100-μm (green) and 10-μm (blue) rulers are used as references for measuring the diameters of microvessels and capillaries, respectively. Measured capillaries are indicated by blue arrows.

Supplementary Figure S3. Pregnancy outcome and the prostacyclin-axis metabolite. (A) Number of miscarriages; (B) peak uterine contraction force; (C) uterine contraction frequency; (D) serum 6-keto-PGF_1α_ (the stable metabolite of prostacyclin). All p-values are shown as numbers, compared with the model group. n = 9 for (A), n = 3 for (B-D).

Supplementary Figure S4. GO and Reactome pathway enrichment analyses of the common targets of QBW, RSA, and PTS. (A) Top 5 enriched GO biological processes; (B) top 5 enriched GO cellular components; (C) top 5 enriched GO molecular functions; (D) top 5 enriched Reactome pathways.

Supplementary Figure S5. Network toxicology analysis of QBW in the liver. (A) TissueEnrich analysis of the predicted toxic targets of QBW; (B) tissue-specific genes enriched in the liver (TissueEnrich); (C) Venn diagram of the intersection between QBW-predicted liver-specific toxic targets and hepatotoxicity targets retrieved from the databases; (D) ingredient-compound-target network of hepatotoxicity; (E) top 20 genes ranked by degree in the ingredient-compound-target network; (F) protein-protein interaction network of hepatotoxicity; (G) top 20 genes ranked by degree in the protein-protein interaction network; (H) top 5 enriched GO biological processes; (I) top 5 enriched GO cellular components; (J) top 5 enriched GO molecular functions; (K) top 5 enriched Reactome pathways.

Supplementary Figure S6. Schematic illustration of the dual-hit RSA with PTS rat model and pharmacological interventions.

Supplementary Discussion S1. Physiological basis of the microvessel-capillary distinction

Microvessels (10-100 μm in diameter), including small arterioles and venules, help regulate blood flow and vascular resistance.^1^ Structural changes in microvessels may signal blood stasis, endothelial dysfunction, or local procoagulant conditions, suggesting impaired uteroplacental perfusion that secondarily affects normal capillary function. Capillaries (<10 μm), on the other hand, serve as the main sites for oxygen and nutrient exchange.^1^ Changes in capillary density may indicate impaired angiogenesis and reduced oxygen delivery within the decidua, posing risks to the fetus in utero.^2^

Supplementary Discussion S2. Anti-inflammatory mechanisms of the major QBW compounds

Both quercetin and kaempferol have been reported to reduce circulating levels of IL-1β and TNF-α. Quercetin modulated inflammatory responses via NF-κB signaling, while kaempferol inhibited NF-κB activation and downregulated PTGS2 expression, leading to reduced prostaglandin production.^3-5^ Given that PTGS2-derived prostaglandins are key mediators of uterine contraction, these effects may contribute to the improved uterine stability observed in QBW-treated rats.

Supplementary Discussion S3. Clinical evidence on low-dose aspirin in recurrent pregnancy loss

Low-dose aspirin is recommended by NICE, WHO, and ACCP for preeclampsia prevention and antiphospholipid syndrome-related recurrent miscarriage management, though its optimality for all RSA subtypes remains uncertain.^6^ Landmark trials, including the ALIFE study, and a Cochrane systematic review demonstrated that aspirin alone did not improve live-birth rates in unexplained or antiphospholipid antibody-associated recurrent pregnancy loss.^7-9^ Emerging evidence also suggests that the standard 75 mg dose may not achieve adequate thromboxane suppression in all pregnant women, with 150 mg providing more consistent platelet inhibition.^10^ Collectively, the limited efficacy of aspirin in our model likely reflects the non-thrombotic pathological drive and potential underdosing rather than a failure of antiplatelet therapy per se.

Supplementary Discussion S4. Hepatic safety: pathway-level protection versus metabolic liability

Network toxicology analysis flagged hepatic targets, although no observable toxicity was detected in the treated animals, as organ weights, gross pathology, and mortality remained unchanged, suggesting that the experimental duration or doses were insufficient to elicit overt toxicity, or that the predicted interactions are mitigated *in vivo.* TissueEnrich analysis showed that the liver-enriched targets, including *CYP3A4*, *CYP7A1*, *NR1H4*, *APOA1*, and *SULT2A1*, cluster around xenobiotic metabolism, bile acid regulation, cholesterol transport, and steroid turnover, indicating increased hepatic metabolic engagement rather than direct injury. This interpretation aligns with the known hepatoprotective activities of the main QBW compounds: kaempferol supports redox and bile-acid homeostasis, and β-sitosterol attenuates steatosis and inflammatory liver injury via sterol and bile-acid pathways.^11-13^ At the same time, quercetin phase II metabolites accumulate through glucuronidation and sulfation routes that overlap with the UGT/SULT/CYP nodes highlighted above, suggesting a maternal metabolic processing burden, particularly during co-administration with aspirin.^14,15^ Given that RSA patients may concurrently receive heparin, progesterone supplementation, or immunomodulatory therapies, potential herb-drug interactions warrant careful consideration in future translational studies. Furthermore, the relatively short experimental duration precludes detection of cumulative hepatic toxicity, and long-term safety assessments with histopathological evaluation remain necessary before clinical application.

Supplementary Discussion S5. Compound-level evidence for the reproductive safety window

Quercetin and its conjugated metabolites cross the placenta, directly supporting the functional relevance of the placental *CYP19A1* axis.^16^ Kaempferol modulates steroid-responsive uterine gene programs and improves trophoblast and placental function in RSA models, providing tissue-level support for both *ESR1*-associated and *CYP19A1*-associated predictions.^17,18^ By contrast, β-sitosterol remains a dose-sensitive plausibility signal in pregnancy, with its effects more likely mediated through maternal-placental sterol handling than through direct uterine or placental activity.^19^

Supplementary Discussion S6. Comparison with classical stress-induced pregnancy-loss models

Psychological stress has been associated with miscarriage in clinical studies,^20,21^ but the present model differs from classical stress-induced pregnancy-loss models in two key respects. First, pregnancy was established before model induction, distinguishing it from models that primarily impair implantation and decidualization.^22^ Second, although sympathetic stimulation can suppress ovarian estradiol secretion and alter progesterone metabolism,^23,24^ the dominant phenotype of the present model involved coagulation imbalance, platelet activation, and microcirculatory dysfunction, with partial aspirin responsiveness confirming a PTS-centered mechanism. The increased uterine contractility further contrasts with the β-adrenergic tocolytic effect of epinephrine in the gravid uterus.^25^ Thus, the model comprises two partially overlapping components, and PTS, although highly prevalent among RSA patients, acts as a contributing factor rather than an independent cause of pregnancy loss; its convergence with progesterone insufficiency creates the high-risk scenario that this dual-hit model aims to capture.^26,27^

Reference:

1.	Munoz CJ, Lucas A, Williams AT, Cabrales P. A Review on Microvascular Hemodynamics: The Control of Blood Flow Distribution and Tissue Oxygenation. *Crit Care Clin.* 2020;36(2):293-305.

2.	Guven G, Hilty MP, Ince C. Microcirculation: Physiology, Pathophysiology, and Clinical Application. *Blood Purif.* 2020;49(1-2):143-150.

3.	Chekalina N, Burmak Y, Petrov Y, et al. Quercetin reduces the transcriptional activity of NF-kB in stable coronary artery disease. *Indian Heart J.* 2018;70(5):593-597.

4.	Basu A, Das AS, Sharma M, et al. STAT3 and NF-kappaB are common targets for kaempferol-mediated attenuation of COX-2 expression in IL-6-induced macrophages and carrageenan-induced mouse paw edema. *Biochem Biophys Rep.* 2017;12:54-61.

5.	Wall C, Lim R, Poljak M, Lappas M. Dietary flavonoids as therapeutics for preterm birth: luteolin and kaempferol suppress inflammation in human gestational tissues in vitro. *Oxid Med Cell Longev.* 2013;2013:485201.

6.	Ozturk O, Greaves M, Templeton A. Aspirin dilemma. Remodelling the hypothesis from a fertility perspective. *Hum Reprod.* 2002;17(5):1146-1148.

7.	Hamulyak EN, Scheres LJ, Marijnen MC, Goddijn M, Middeldorp S. Aspirin or heparin or both for improving pregnancy outcomes in women with persistent antiphospholipid antibodies and recurrent pregnancy loss. *Cochrane Database Syst Rev.* 2020;5(5):CD012852.

8.	Kaandorp SP, Goddijn M, van der Post JA, et al. Aspirin plus heparin or aspirin alone in women with recurrent miscarriage. *N Engl J Med.* 2010;362(17):1586-1596.

9.	Kaandorp SP, Goddijn M, Post JAMvd, et al. Aspirin and Aspirin Combined with Low-Molecular-Weight Heparin in Women with Unexplained Recurrent Miscarriage: a Randomized Controlled Multicenter Trial (ALIFE Study). *Blood.* 2009;114(22):488.

10.	Vinogradov R, Kavanagh ON, Palmer J, et al. A randomized crossover design study comparing the pharmacokinetics and pharmacodynamics of 2 single doses of oral aspirin (75 mg v 150 mg) in pregnant women at risk of preeclampsia: implications on assessing aspirin response and patient adherence to therapy. *Am J Obstet Gynecol.* 2025;232(5):474 e471-474 e415.

11.	Niziński P, Krajewska A, Oniszczuk T, Polak B, Oniszczuk A. Hepatoprotective Effect of Kaempferol-A Review. *Molecules.* 2025;30(9):1913.

12.	Abo-Zaid OA, Moawed FS, Ismail ES, Farrag MA. β-sitosterol attenuates high- fat diet-induced hepatic steatosis in rats by modulating lipid metabolism, inflammation and ER stress pathway. *BMC Pharmacol Toxicol.* 2023;24(1):31.

13.	Chen Z, Wu A, Jin H, Liu F. β-Sitosterol attenuates liver injury in a rat model of chronic alcohol intake. *Arch Pharm Res.* 2020;43(11):1197-1206.

14.	Tang L, Zhou J, Yang CH, Xia BJ, Hu M, Liu ZQ. Systematic studies of sulfation and glucuronidation of 12 flavonoids in the mouse liver S9 fraction reveal both unique and shared positional preferences. *J Agric Food Chem.* 2012;60(12):3223-3233.

15.	Kuehl GE, Bigler J, Potter JD, Lampe JW. Glucuronidation of the aspirin metabolite salicylic acid by expressed UDP-glucuronosyltransferases and human liver microsomes. *Drug Metab Dispos.* 2006;34(2):199-202.

16.	Fujiwara N, Matsubara K, Mukai R, et al. Placental transfer of quercetin and its metabolites to fetuses in pregnant mice. *Biochem Biophys Res Commun.* 2025;788:152827.

17.	Bergsten TM, Li K, Lantvit DD, Murphy BT, Burdette JE. Kaempferol, a Phytoprogestin, Induces a Subset of Progesterone-Regulated Genes in the Uterus. *Nutrients.* 2023;15(6).

18.	Xu G, Li H, Fang L, et al. Kaempferol improves recurrent spontaneous abortion outcomes and prevents oxidative stress damage by inhibiting the NF-kB signaling pathway. *Food Chem Toxicol.* 2025;202:115540.

19.	Furukawa S, Hayashi S, Usuda K, Abe M, Hagio S, Ogawa I. Toxicological pathology in the rat placenta. *J Toxicol Pathol.* 2011;24(2):95-111.

20.	Qu F, Wu Y, Zhu YH, et al. The association between psychological stress and miscarriage: A systematic review and meta-analysis. *Sci Rep.* 2017;7(1):1731.

21.	Anastasiadou E, Bacopoulou F, Vlachakis D, Critselis E, Kanaka-Gantenbein C. The Association Between Work-Related Stress and Miscarriage in Pregnant Women: A Systematic Review. *Adv Exp Med Biol.* 2026;1489:169-174.

22.	Wu J, Kong S, Guo C, et al. An exaggerated epinephrine-adrenergic receptor signaling impairs uterine decidualization in mice. *Reprod Toxicol.* 2019;90:109-117.

23.	Uchida S. Sympathetic regulation of estradiol secretion from the ovary. *Auton Neurosci.* 2015;187:27-35.

24.	Meyer SG. Adrenergic influence on progesterone metabolism and cyclicity in the rat ovary: autotransplantation and chemical sympathectomy. *J Steroid Biochem Mol Biol.* 1992;43(8):885-894.

25.	Segal S, Csavoy AN, Datta S. The tocolytic effect of catecholamines in the gravid rat uterus. *Anesth Analg.* 1998;87(4):864-869.

26.	Sarig G, Younis JS, Hoffman R, Lanir N, Blumenfeld Z, Brenner B. Thrombophilia is common in women with idiopathic pregnancy loss and is associated with late pregnancy wastage. *Fertil Steril.* 2002;77(2):342-347.

27.	Zheng W, Zhang L, Ning J, et al. Recent research progress on pathophysiological mechanism and clinical treatment of recurrent spontaneous abortion and prethrombotic state. *J Reprod Immunol.* 2026;173:104822.
